# Supplementary material for: Cardiometabolic indices as predictors of clinical outcomes in palliative care patients
Source: PLoS One. 2026 May 8;21(5):e0338562. doi: 10.1371/journal.pone.0338562 (PMC13155664; doi:10.1371/journal.pone.0338562)
Supplement: S1 File — (DOCX) [file pone.0338562.s001.docx]

# Graphical Abstract

| **STUDY DESIGN**  Retrospective Cohort: 318 Palliative Care Patients (2014-2025)  ▼  **12 CARDIOMETABOLIC INDICES COMPARED**   \| **Metabolic**  **TyG-BMI** \| **Lipid-Based**  AIP • CRI-I/II  Non-HDL • TG/HDL \| **Inflammatory**  NLR • PLR • SII • MHR  CAR • PNI \| \| --- \| --- \| --- \|   ▼  **PRIMARY OUTCOMES**  **Sepsis • Mechanical Ventilation • 30-Day Mortality (9.4%)**  ▼  **KEY FINDINGS**   \| **TyG-BMI SUPERIORITY**  **Sepsis AUC: 0.84**  **Mortality AUC: 0.87**  vs. Others: 0.62-0.78 \| **DIABETIC PATIENTS**  Enhanced Performance  **AUC: 0.92 (0.87-0.97)**  **OR: 2.65 vs 1.95** \| \| --- \| --- \|   **CONCLUSION**  **TyG-BMI outperforms all traditional indices with exceptional discriminative ability in diabetic patients, supporting its prioritization for risk stratification in palliative care.** |
| --- | --- | --- | --- | --- | --- |
