# Supplementary material for: Cardiometabolic indices as predictors of clinical outcomes in palliative care patients
Source: PLoS One. 2026 May 8;21(5):e0338562. doi: 10.1371/journal.pone.0338562 (PMC13155664; doi:10.1371/journal.pone.0338562)
Supplement: Attachment — Submitted filename: Response_to_Reviewers_PLOS_ONE 01.02.docx [file pone.0338562.s003.docx]

**RESPONSE TO REVIEWERS**

**Dear Editor and Reviewers,**

We are deeply grateful for the thorough and constructive evaluation of our manuscript by the Editor and both Reviewers. The insightful comments and valuable suggestions have significantly contributed to improving the scientific rigor, clarity, and overall quality of our work. We have carefully addressed each point raised by the reviewers and have made substantial revisions to the manuscript accordingly. The revised text additions are indicated with quotation marks ('') throughout this response and in the manuscript. Below, we provide a point-by-point response to all comments.

# JOURNAL REQUIREMENTS AND EDITOR COMMENTS

### Requirement 1: PLOS ONE Style Requirements

**Comment:** Please ensure that your manuscript meets PLOS ONE's style requirements, including those for file naming.

**Response:** We sincerely thank the Editor for this guidance. We have carefully reviewed the PLOS ONE formatting templates and have revised our manuscript to ensure full compliance with the journal's style requirements. The manuscript formatting, including title page, author affiliations, headings, references, and figure/table formatting, has been updated according to the provided templates. File naming conventions have also been corrected to meet journal specifications.

### Requirement 2: Data Availability

**Comment:** Please address data sharing restrictions and provide appropriate data access information.

**Response:** We appreciate this important clarification request. There are ethical restrictions on sharing the complete dataset publicly due to the sensitive nature of patient information in palliative care settings. Specifically, the data contain potentially identifying patient information including detailed clinical histories, comorbidity profiles, and outcome data from a relatively small, vulnerable population (n=318). The Ethics Committee of Yildirim Beyazit University Yenimahalle Training and Research Hospital, which approved this study, has imposed restrictions on public data sharing to protect patient privacy and confidentiality. We have revised the Data Availability Statement as follows:

*'Data cannot be shared publicly due to ethical restrictions imposed by the Ethics Committee of Yildirim Beyazit University Yenimahalle Training and Research Hospital to protect patient privacy. The dataset contains sensitive clinical information from a vulnerable palliative care population. Data access requests may be submitted to the Ethics Committee (Contact: Yildirim Beyazit University Yenimahalle Training and Research Hospital Ethics Committee, Ankara, Turkey; Email: yikiletikkurul@ybu.edu.tr) for researchers who meet the criteria for access to confidential data.'*

### Requirement 3: Ethics Statement Placement

**Comment:** Your ethics statement should only appear in the Methods section.

**Response:** We thank the Editor for this clarification. We have ensured that the ethics statement appears only in the Methods section. Any duplicate ethics information in other sections has been removed. The ethics statement now reads in the Methods section:

*'The Institutional Ethics Committee of Etimesgut Şehit Sait Ertürk State Hospital approved this study with waived informed consent given the retrospective design and use of anonymized data. All procedures complied with the ethical standards of the institutional and national research committees and with the 2013 revision of the Declaration of Helsinki.'*

### Requirement 4: Citation of Recommended Works

**Comment:** If the reviewer comments include a recommendation to cite specific previously published works, please review and evaluate these publications.

**Response:** We appreciate this guidance. We have carefully reviewed the reviewer comments and no specific citation recommendations were made. We have ensured that our reference list comprehensively covers the relevant literature supporting our methodology and findings.

# REVIEWER #1 COMMENTS AND RESPONSES

### Comment 1.1: Overfitting and Events-Per-Variable Ratio

**Comment:** Only 30 patients died (9.4%), yet the regression models include many covariates. This yields a very low events-per-variable ratio (~3 events per variable), which risks overfitting and unstable estimates. The authors should acknowledge the limited events and consider simplifying models or using penalized regression.

**Response:** We sincerely thank the Reviewer for this critical and methodologically important observation. The concern regarding the events-per-variable (EPV) ratio is entirely valid and represents a fundamental consideration in logistic regression modeling. We have carefully reconsidered our analytical approach and have made the following revisions:

First, we acknowledge that the traditional EPV rule of 10 events per predictor variable is often recommended to ensure stable coefficient estimates. With 30 mortality events and multiple covariates in our original models, this threshold was not met, potentially leading to overfitted models with inflated odds ratios and wide confidence intervals.

Second, we have revised our multivariable analysis approach by implementing simplified, parsimonious models. The revised primary analysis includes only clinically essential covariates: age, sex, and primary diagnosis category, reducing the number of predictors while maintaining clinical relevance.

Third, we have added the following explicit acknowledgment in the Limitations section:

*'The limited number of mortality events (n=30) relative to the number of covariates in multivariable models yields a low events-per-variable ratio, which may result in overfitting and potentially unstable odds ratio estimates. Although we employed parsimonious models to mitigate this concern, these findings require validation in larger cohorts with higher event rates to confirm the robustness of the reported associations.'*

### Comment 1.2: Adjustment for Diabetes as Covariate

**Comment:** TyG-BMI is inherently related to glucose metabolism and thus to diabetes. Adjusting for diabetes may attenuate TyG-BMI's effect or introduce multicollinearity. It may be more appropriate to present models without including diabetes as a covariate when evaluating TyG-BMI.

**Response:** We greatly appreciate this insightful methodological comment. The Reviewer raises an excellent point regarding the potential for multicollinearity and effect attenuation when adjusting for diabetes in models evaluating TyG-BMI. We have made the following modifications:

We now present our primary multivariable models without diabetes as a covariate, as suggested. This approach allows the full prognostic information captured by TyG-BMI to be evaluated without potential attenuation from collinear adjustment. We have added the following clarification to the Methods section:

*'Given the inherent relationship between TyG-BMI and glucose metabolism, diabetes mellitus was not included as a covariate in the primary multivariable models to avoid potential multicollinearity and effect attenuation. Instead, diabetes status was evaluated as an effect modifier through stratified analyses and formal interaction testing.'*

Additionally, we have clarified in the Discussion section that the stratified analysis by diabetes status serves as the primary means of evaluating differential performance, rather than including diabetes as a covariate in pooled models.

### Comment 1.3: Clinical Significance of AUC Differences

**Comment:** TyG-BMI had higher AUCs (e.g. 0.87 vs 0.78 for mortality), with p<0.001. While statistically significant, the practical importance of these differences should be addressed. The authors describe this as a "clinically meaningful improvement", but they should explain how this translates into patient care or decision-making.

**Response:** We sincerely appreciate this thoughtful comment. The Reviewer correctly emphasizes that statistical significance alone does not establish clinical relevance. We have substantially revised our discussion of AUC differences to provide a more balanced and clinically contextualized interpretation:

We have added the following paragraph to the Discussion section to address practical clinical implications:

*'The clinical significance of an AUC improvement from 0.78 to 0.87 warrants careful consideration. According to established guidelines for interpreting discriminative ability, AUC values between 0.80 and 0.90 represent "good" discrimination, while values exceeding 0.90 indicate "excellent" discrimination. The observed 0.09-unit AUC improvement translates to a meaningful enhancement in risk stratification: at the optimal TyG-BMI threshold, the positive likelihood ratio increased from 3.2 (for AIP) to 4.6, indicating substantially stronger predictive capability. In practical terms, a palliative care patient with TyG-BMI above the threshold has approximately 4.6-fold higher odds of 30-day mortality compared to those below the threshold. This magnitude of improvement may facilitate more confident goals-of-care discussions and resource allocation decisions. However, we acknowledge that the ultimate clinical utility of any biomarker depends on whether its use leads to improved patient outcomes, which requires prospective interventional validation.'*

### Comment 1.4: Cut-off Values and External Validation

**Comment:** Your paper identifies TyG-BMI cut-offs (220 for mortality, 235 for sepsis) based on Youden's index in this sample. These thresholds are data-driven and likely overfit. I recommend softening language (e.g. "potentially useful thresholds") and emphasizing validation.

**Response:** We are grateful for this important guidance regarding the interpretation of data-derived cut-offs. We fully agree that Youden's index-derived thresholds are sample-specific and may not generalize to external populations. We have made the following changes:

We have softened the language throughout the manuscript when referring to cut-off values. The revised text now states:

*'The Youden's index-derived cut-off values of 220 for mortality and 235 for sepsis represent potentially useful thresholds for hypothesis generation; however, these data-driven values are sample-specific and require external validation before clinical implementation. Given the exploratory nature of these cut-offs and the potential for optimism bias inherent in single-cohort derivation, prospective validation in independent populations is essential before these thresholds can be recommended for routine clinical use.'*

We have also emphasized external validation as a critical future research priority in both the Discussion and Conclusion sections.

### Comment 1.5: Comparison of Odds Ratios

**Comment:** The text comments that TyG-BMI's OR is "50%" higher than AIP's OR (2.38 vs 1.58). This relative comparison of ORs across models can be misleading. It might be clearer to report that TyG-BMI had a larger effect size than any other index, without using percentages of ratios.

**Response:** We thank the Reviewer for this methodologically sound suggestion. We agree that comparing odds ratios using percentage differences can be misleading, particularly when comparing indices with different distributions and scales. We have revised the relevant text as follows:

*'TyG-BMI demonstrated the largest effect size among all evaluated indices (OR 2.38 per SD, 95% CI 1.78–3.18), substantially exceeding the next-best performing index, AIP (OR 1.58, 95% CI 1.18–2.12). The non-overlapping confidence intervals indicate a meaningful difference in prognostic strength between these indices.'*

We have removed all percentage-based comparisons of odds ratios from the manuscript and instead focus on effect sizes with confidence intervals, allowing readers to judge the magnitude of differences directly.

### Comment 1.6: Mortality Rate Typo in Discussion

**Comment:** In Results, 30-day mortality is 30 patients (9.4%), but the Discussion incorrectly refers to a "30-day mortality rate of 30%". This should be corrected.

**Response:** We sincerely apologize for this typographical error and thank the Reviewer for identifying it. This was indeed a significant error that could mislead readers. We have corrected the Discussion section to accurately state:

*'The 30-day mortality rate of 9.4% (30 patients) in our palliative cohort reflects the overall risk profile of this population.'*

### Comment 1.7: Speculative Mechanistic Explanations

**Comment:** The Discussion provides a strong rationale for TyG-BMI's performance. While plausible, these explanations are speculative. The authors should clarify that mechanistic links are hypothetical given observational data.

**Response:** We appreciate this important reminder about the distinction between observed associations and mechanistic causation. We have revised the Discussion to more clearly frame mechanistic explanations as hypothetical. The revised text now includes:

*'Several potential mechanisms may explain TyG-BMI's superior prognostic performance, although these proposed pathways remain hypothetical given the observational nature of our study. The biological plausibility of these associations warrants discussion, with the understanding that causal inference cannot be established from retrospective data. TyG-BMI may capture metabolic dysregulation through its integration of insulin resistance markers and adiposity; however, definitive mechanistic links require prospective studies with appropriate biomarker measurements and potential interventional designs.'*

### Comment 1.8: Exclusion Criteria and Selection Bias

**Comment:** The manuscript does not quantify how many were excluded or assess how this might bias results.

**Response:** We thank the Reviewer for highlighting this important methodological detail. We have added a patient flow description to the Methods section and a corresponding flow diagram (Figure S1) to transparently report exclusions. The revised text now includes:

*'During the study period, 412 patients were admitted to the palliative care unit. Of these, 94 patients (22.8%) were excluded: 67 due to incomplete laboratory data required for index calculation (primarily missing lipid profiles), 18 due to incomplete 30-day follow-up data, and 9 due to age <18 years. The final analytical cohort comprised 318 patients (77.2% of screened population). Patients excluded for missing laboratory data were similar in age and sex distribution to included patients, although they had shorter median length of stay, suggesting that excluded patients may have had more rapidly progressive illness or earlier discharge/death, which could introduce selection bias toward patients with more complete clinical workup.'*

### Comment 1.9: Interpretation Tone

**Comment:** Since it is a single center study with limited population and so many variables to be included; your interpretation should be more cautious.

**Response:** We sincerely appreciate this guidance and acknowledge that our original tone may have been overly assertive given the study's limitations. We have comprehensively revised the manuscript to adopt a more cautious and appropriately measured interpretive stance. Specifically:

We have changed definitive statements to conditional language throughout the manuscript. The revised Conclusion now reads:

*'In this single-center retrospective cohort, TyG-BMI demonstrated potentially superior prognostic performance compared to established cardiometabolic indices for predicting sepsis and 30-day mortality in palliative care patients. These preliminary findings suggest that TyG-BMI may be a promising biomarker warranting further investigation, particularly in diabetic patients where enhanced discriminative ability was observed. However, given the study limitations including the single-center design, relatively small sample size, and data-derived cut-offs, these findings require validation in prospective, multicenter studies before clinical implementation can be recommended.'*

# REVIEWER #2 COMMENTS AND RESPONSES

### Comment 2.1: Specification of Units in Index Formulas

**Comment:** While the formulas for the cardiometabolic indices are clearly presented, units are not consistently specified for all components. Explicitly stating units for each formula would improve reproducibility and facilitate external validation.

**Response:** We greatly appreciate this constructive suggestion for improving reproducibility. We have revised the Cardiometabolic Index Calculation section to explicitly specify units for all formula components. The revised Methods section now includes:

*'Twelve cardiometabolic indices were calculated using standard formulas with the following specified units: Primary Index: TyG-BMI: ln[TG (mg/dL) × FPG (mg/dL) / 2] × BMI (kg/m²) Lipid-Based Indices: AIP: Log₁₀[TG (mg/dL) / HDL-C (mg/dL)] CRI-I: Total Cholesterol (mg/dL) / HDL-C (mg/dL) CRI-II: LDL-C (mg/dL) / HDL-C (mg/dL) Non-HDL: Total Cholesterol (mg/dL) − HDL-C (mg/dL) TG/HDL: TG (mg/dL) / HDL-C (mg/dL) Inflammatory Indices: NLR: Neutrophil count (×10⁹/L) / Lymphocyte count (×10⁹/L) PLR: Platelet count (×10⁹/L) / Lymphocyte count (×10⁹/L) SII: [Platelet count (×10⁹/L) × Neutrophil count (×10⁹/L)] / Lymphocyte count (×10⁹/L) MHR: Monocyte count (×10⁹/L) / HDL-C (mmol/L) Nutritional/Integrated Indices: PNI: Albumin (g/L) + [5 × Lymphocyte count (×10⁹/L)] CAR: CRP (mg/L) / Albumin (g/L)'*

### Comment 2.2: Redundancy and Overlap Among Indices

**Comment:** Several of the compared indices share overlapping biological or mathematical components. Acknowledging this overlap in the Discussion would help readers interpret why certain indices perform similarly.

**Response:** We thank the Reviewer for this insightful observation. We have added a new paragraph to the Discussion section addressing component overlap and its implications for interpretation:

*'It is important to acknowledge that several evaluated indices share overlapping biological or mathematical components, which may explain similar performance patterns within index categories. Among lipid-based indices, AIP, TG/HDL ratio, and CRI indices all incorporate HDL-cholesterol as a denominator, reflecting shared capture of atherogenic dyslipidemia. Similarly, inflammatory indices (NLR, PLR, SII) share lymphocyte count as a common component, while SII additionally incorporates elements of both NLR and PLR. This mathematical overlap creates inherent correlation among indices within categories and partially explains their comparable discriminative abilities. TyG-BMI's distinct advantage may derive from its integration of components (glucose, triglycerides, BMI) that are not shared with purely lipid-based or inflammatory indices, thereby capturing complementary pathophysiological information.'*

### Comment 2.3: Adjustment for Acute Illness Severity

**Comment:** Additional markers of acute illness severity (e.g., organ dysfunction scores, ICU-level interventions) may not have been uniformly available. Please acknowledge this limitation and discuss the possibility that TyG-BMI may partly reflect acute physiological stress at admission.

**Response:** We appreciate this important methodological consideration. We have added explicit acknowledgment of this limitation and its potential implications. The revised Limitations section now includes:

*'Our multivariable models did not include standardized markers of acute illness severity such as Sequential Organ Failure Assessment (SOFA) scores, Acute Physiology and Chronic Health Evaluation (APACHE) scores, or documentation of ICU-level interventions, as these were not uniformly documented in our retrospective palliative care dataset. This limitation is notable because acute physiological stress at admission can independently affect both TyG-BMI components (stress hyperglycemia, lipid mobilization) and clinical outcomes. Therefore, the observed associations between TyG-BMI and adverse outcomes may partly reflect acute illness severity rather than baseline metabolic risk alone. Future prospective studies incorporating validated illness severity scores would help disentangle the contributions of chronic metabolic dysfunction versus acute stress-related metabolic derangements to TyG-BMI's prognostic value.'*

### Comment 2.4: Interpretation of ROC-Derived Cut-offs

**Comment:** The language describing cut-offs as "actionable" could be tempered and framed as hypothesis-generating pending validation in independent cohorts.

**Response:** We agree with this recommendation and have revised the language throughout the manuscript. The term "actionable" has been replaced with more appropriate terminology. The revised text now states:

*'The identified TyG-BMI cut-offs of 220 for mortality prediction and 235 for sepsis prediction should be considered hypothesis-generating values derived from this specific cohort. While these thresholds demonstrate promising test characteristics within our sample (sensitivity 83%, specificity 82% for mortality), they require rigorous external validation in independent populations before any consideration for clinical decision-making. Until validated, these cut-offs serve primarily to inform the design of future prospective studies and should not be implemented in routine clinical practice.'*

### Comment 2.5: Mechanical Ventilation as an Outcome

**Comment:** In palliative care settings, the decision to initiate mechanical ventilation may be influenced by goals-of-care discussions and institutional practice in addition to disease severity. Clarifying this context would strengthen the manuscript.

**Response:** We greatly appreciate this nuanced observation regarding the complexity of mechanical ventilation decisions in palliative care contexts. We have added the following clarification to both the Methods and Discussion sections:

In the Methods section:

*'Requirement for invasive mechanical ventilation was documented when patients were intubated and connected to mechanical ventilatory support during their hospitalization. It should be noted that in palliative care settings, the decision to initiate mechanical ventilation is influenced by multiple factors beyond disease severity alone, including advance directives, goals-of-care discussions, family preferences, and institutional practices. Therefore, this outcome reflects a combination of clinical deterioration and treatment preference considerations.'*

In the Discussion section:

*'The interpretation of mechanical ventilation as an outcome requires acknowledgment of the palliative care context. Unlike in acute care settings where mechanical ventilation primarily reflects disease severity requiring life support, the initiation of mechanical ventilation in palliative care is substantially influenced by pre-established goals-of-care, advance care planning, and shared decision-making between patients, families, and care teams. Consequently, TyG-BMI's association with mechanical ventilation may reflect both its capacity to identify patients at risk for respiratory deterioration and potential confounding by patient and family preferences for aggressive interventions. Future studies should consider stratifying analyses by documented code status or incorporating goals-of-care variables as covariates.'*

### Comment 2.6: Fasting Status of Laboratory Measurements

**Comment:** The TyG index assumes fasting glucose and triglyceride measurements. Given the palliative care context, where true fasting conditions may not always be feasible, a short clarification would be helpful.

**Response:** We thank the Reviewer for raising this important practical consideration that is particularly relevant to palliative care populations. We have added the following clarification to the Methods and Limitations sections:

In the Methods section:

*'Laboratory parameters were obtained from blood samples collected during the first 24 hours of admission as part of routine clinical care. While the TyG index formula assumes fasting glucose and triglyceride measurements, true fasting conditions were not systematically verified or documented in our retrospective dataset. Standard institutional protocol recommends morning fasting blood draws; however, in palliative care settings, strict fasting requirements may be relaxed based on patient comfort and clinical priorities.'*

In the Limitations section:

*'The TyG index calculation assumes fasting glucose and triglyceride values; however, true fasting status could not be verified in this retrospective cohort. Non-fasting samples would be expected to yield higher glucose and triglyceride values, potentially introducing measurement variability and affecting index calculation accuracy. In palliative care settings, where patient comfort often takes precedence over strict adherence to fasting protocols, this source of variability may be particularly relevant. Reassuringly, recent evidence suggests that TyG-related indices retain prognostic value even when calculated from non-fasting samples, although the absolute cut-off values may differ. Future prospective studies should document fasting status to evaluate its impact on TyG-BMI performance.'*

# SUMMARY OF REVISIONS

In summary, we have made the following major revisions to address reviewer concerns:

- Simplified multivariable models and acknowledged EPV limitations
- Removed diabetes as covariate from primary TyG-BMI models
- Provided clinical context for AUC differences
- Softened language regarding cut-off values
- Corrected mortality rate typo (9.4%, not 30%)
- Added patient flow diagram and exclusion details
- Specified units for all index formulas
- Discussed index component overlap
- Acknowledged lack of illness severity adjustment
- Clarified mechanical ventilation context in palliative care
- Addressed fasting status uncertainty
- Updated Data Availability Statement
- Adopted more cautious interpretive tone throughout

We are sincerely grateful to both Reviewers for their thorough and constructive evaluation. Their expert feedback has substantially improved the scientific rigor, transparency, and clinical relevance of our manuscript. We believe that the revised manuscript addresses all concerns raised and is now suitable for publication in PLOS ONE.

Respectfully submitted,

**Mete Ucdal, MD**

On behalf of all authors
